# Effect of a reduced fat and sugar maternal dietary intervention during lactation on the infant gut microbiome

**DOI:** 10.3389/fmicb.2022.900702

**Published:** 2022-08-17

**Authors:** Azhar S. Sindi, Lisa F. Stinson, Soo Sum Lean, Yit-Heng Chooi, Gabriela E. Leghi, Merryn J. Netting, Mary E. Wlodek, Beverly S. Muhlhausler, Donna T. Geddes, Matthew S. Payne

**Affiliations:** ^1^Division of Obstetrics and Gynecology, The University of Western Australia, Perth, WA, Australia; ^2^College of Applied Medical Sciences, Umm Al-Qura University, Makkah, Saudi Arabia; ^3^School of Molecular Sciences, The University of Western Australia, Perth, WA, Australia; ^4^School of Agriculture, Food and Wine, The University of Adelaide, Adelaide, SA, Australia; ^5^Women and Kids Theme, South Australian Health and Medical Research Institute (SAHMRI), Adelaide, SA, Australia; ^6^Discipline of Pediatrics, The University of Adelaide, Adelaide, SA, Australia; ^7^Women’s and Children’s Hospital, Adelaide, SA, Australia; ^8^Department of Obstetrics and Gynecology, University of Melbourne, Melbourne, VIC, Australia; ^9^CSIRO, Adelaide, SA, Australia; ^10^Women and Infants Research Foundation, Perth, WA, Australia

**Keywords:** maternal diet, breastfeeding, breast milk, infant gut microbiome, microbial metagenomics, metagenomic sequencing

## Abstract

**Objective:**

A growing body of literature has shown that maternal diet during pregnancy is associated with infant gut bacterial composition. However, whether maternal diet during lactation affects the exclusively breastfed infant gut microbiome remains understudied. This study sets out to determine whether a two-week of a reduced fat and sugar maternal dietary intervention during lactation is associated with changes in the infant gut microbiome composition and function.

**Design:**

Stool samples were collected from four female and six male (*n* = 10) infants immediately before and after the intervention. Maternal baseline diet from healthy mothers aged 22–37 was assessed using 24-h dietary recall. During the 2-week dietary intervention, mothers were provided with meals and their dietary intake was calculated using FoodWorks 10 Software. Shotgun metagenomic sequencing was used to characterize the infant gut microbiome composition and function.

**Results:**

In all but one participant, maternal fat and sugar intake during the intervention were significantly lower than at baseline. The functional capacity of the infant gut microbiome was significantly altered by the intervention, with increased levels of genes associated with 28 bacterial metabolic pathways involved in biosynthesis of vitamins (*p* = 0.003), amino acids (*p* = 0.005), carbohydrates (*p* = 0.01), and fatty acids and lipids (*p* = 0.01). Although the dietary intervention did not affect the bacterial composition of the infant gut microbiome, relative difference in maternal fiber intake was positively associated with increased abundance of genes involved in biosynthesis of storage compounds (*p* = 0.016), such as cyanophycin. Relative difference in maternal protein intake was negatively associated with *Veillonella parvula* (*p* = 0.006), while positively associated with *Klebsiella michiganensis* (*p* = 0.047). Relative difference in maternal sugar intake was positively associated with *Lactobacillus paracasei* (*p* = 0.022). Relative difference in maternal fat intake was positively associated with genes involved in the biosynthesis of storage compounds (*p* = 0.015), fatty acid and lipid (*p* = 0.039), and metabolic regulator (*p* = 0.038) metabolic pathways.

**Conclusion:**

This pilot study demonstrates that a short-term maternal dietary intervention during lactation can significantly alter the functional potential, but not bacterial taxonomy, of the breastfed infant gut microbiome. While the overall diet itself was not able to change the composition of the infant gut microbiome, changes in intakes of maternal protein and sugar during lactation were correlated with changes in the relative abundances of certain bacterial species.

Clinical trial registration: Australian New Zealand Clinical Trials Registry (ACTRN12619000606189).

## Introduction

The early postnatal period is a critical window for the development of the infant gut microbiome (Derrien et al., 2019), which has been associated with the programming of lifelong health and disease risk (Arrieta et al., 2015, 2018; Walker, 2017). The infant gut microbiome has a role in immune system development and protection against colonization with pathogens (Olin et al., 2018). Perturbations to the infant gut microbiota have been associated with the development of chronic diseases, including allergic disorders and obesity (Prescott, 2013). Early life gut microbiome establishment is a relatively dynamic process that is influenced by a range of environmental and host factors, including maternal diet (Chu et al., 2016; Lundgren et al., 2018; Savage et al., 2018; Ponzo et al., 2019; Babakobi et al., 2020; García-Mantrana et al., 2020), mode of delivery (Bäckhed et al., 2015; Bokulich et al., 2016), feeding practices (breastfeeding, formula, and the introduction of solid food; Thompson et al., 2015; Timmerman et al., 2017; Ho et al., 2018; Stewart et al., 2018), antibiotic use (Bokulich et al., 2016), gestational age at delivery (Hill et al., 2017), host genetics (Benson et al., 2010), and geography (De Filippo et al., 2010; Lin et al., 2013). However, breastfeeding has been reported to be the single most important factor associated with infant gut microbiome composition and function (Stewart et al., 2018).

Several studies have investigated maternal contributions to infant health. Maternal diet during pregnancy has been associated with infant health outcomes, including allergic diseases (Chatzi et al., 2008, 2013); diet-associated alterations to the infant gut microbiome may be implicated in such cases. To date, there have been several observational studies investigating the effect of maternal diet during gestation on the infant gut microbiome (Chu et al., 2016; Lundgren et al., 2018; Savage et al., 2018; Ponzo et al., 2019; Babakobi et al., 2020; García-Mantrana et al., 2020). However, only one study has examined the effect of maternal diet during lactation on the infant gut microbiome. Unfortunately, the results of this study are limited by the fact that maternal dietary intake during lactation was combined with maternal dietary intake during pregnancy, so that the effect of the diet during the lactation period alone could not be analyzed (Babakobi et al., 2020). All previous studies examining the relationship between maternal diet and the infant gut microbiome suffer from a number of flaws related to the methods used to assess maternal dietary intake, time of maternal dietary assessment in relation to infant stool sample collection and underreporting of confounders such as antibiotics, vitamin supplements, and probiotics, all of which may impact results. The use of short amplicon sequencing by these studies also generally limits taxonomical resolution of bacterial communities to the genus level (Walsh et al., 2018) and provides no accurate information on their functional potential (Langille et al., 2013). As such, well-designed dietary intervention studies are required to better understand the effect of maternal diet during lactation on the infant gut microbiome.

The most likely mechanisms by which maternal diet affects the infant gut microbiome and for which the most robust evidence exists is the entero-mammary pathway (Jiménez et al., 2008; Jost et al., 2014; Rodríguez, 2014; Milani et al., 2015; Fernández et al., 2016; Asnicar et al., 2017; Duranti et al., 2017; Murphy et al., 2017; Kordy et al., 2020), wherein gut bacteria are transported to the lactating mammary gland and thereby contribute to the human milk (HM) microbiome. It is well established that diet is a key factor that shapes the gut microbiome (De Filippo et al., 2010; Wu et al., 2011; Fava et al., 2012; Yatsunenko et al., 2012; Lin et al., 2013; David et al., 2014; Graf et al., 2015; Kovatcheva-Datchary et al., 2015; De Filippis et al., 2016; Mandal et al., 2016; Röytiö et al., 2017). In addition, several studies have also shown that maternal diet is associated with the HM microbiome (Williams et al., 2017; Padilha et al., 2019; Babakobi et al., 2020; Cortes-Macías et al., 2020; LeMay-Nedjelski et al., 2020). Since the maternal gut is considered one of the sources of microbes for HM, we hypothesized that maternal diet during lactation can influence the infant gut microbiome. Understanding the effect of the maternal diet during lactation on the early life gut microbiome may allow optimization of dietary recommendations for lactating women to better support infant health and development.

The aim of this study was to determine the effect of a 2-week of a reduced fat and sugar maternal dietary intervention on infant gut microbiome composition and function using shotgun metagenomic sequencing in 10 healthy infants.

## Materials and methods

### Participants

Healthy, Caucasian, primiparous mothers with ages ranging from 22 to 37 years were invited to participate in the study (*n* = 10). All infants included in our study were healthy, exclusively breastfed, and aged 1.8–4.9 months. Six of the infants were born vaginally, two by emergency caesarean section, two by elective caesarean section, and four were female. During the study period, there was no consumption of antibiotic by infants and mothers. Exclusion criteria were pre-existing maternal diabetes, maternal diseases known to affect gastric absorption (such as gastric ulcers), dietary restrictions (such as vegan, vegetarian, gluten-free, milk-free, or dairy-free diets), pregnancy complications (including gestational diabetes, preeclampsia, preterm delivery, and foetal growth restriction), multiple pregnancies, known congenital abnormalities or health issues in the infant that could significantly affect feeding behavior, and solid food introduction before the first study session. The study was approved by The University of Western Australia Human Research Ethics Committee (RA/4/20/4953) and registered on the Australian New Zealand Clinical Trials Registry (ACTRN12619000606189). All mothers provided informed consent and answered a background health and lifestyle questionnaire on enrolment.

### Study design

**Figure 1:**
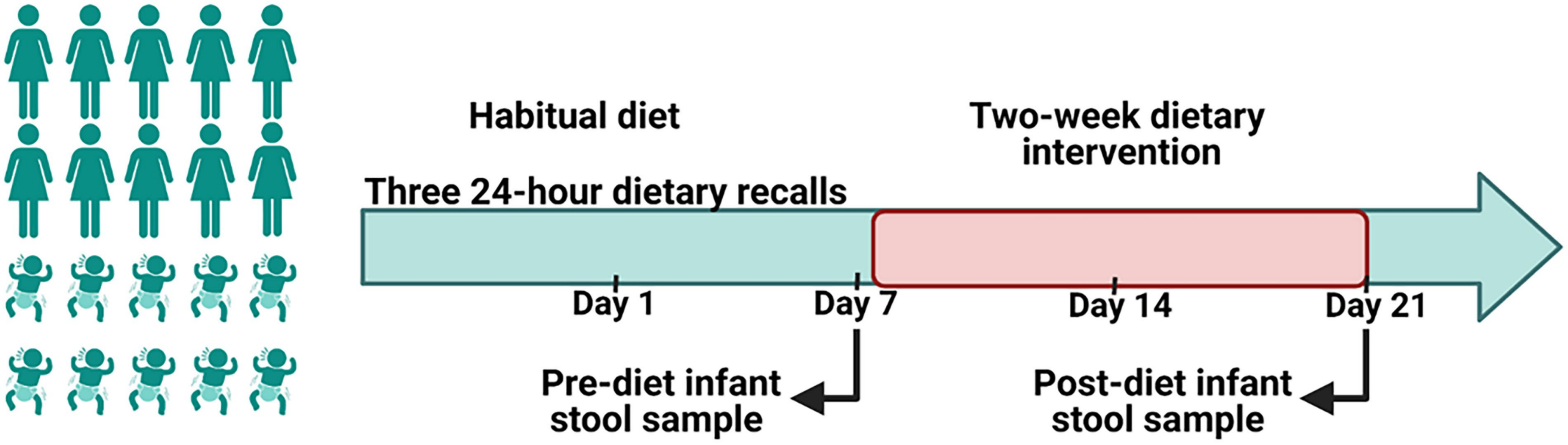
A schematic representation of the study design. A 2-week of a reduced fat and sugar maternal dietary intervention during lactation was performed to evaluate the effect of maternal diet on the infant gut microbiome. Before the intervention, mothers consumed their habitual diet, which was assessed using 24 h dietary recalls. During the intervention maternal dietary intake was analyzed using FoodWorks 10; Xyris Software. Infant stool samples were collected immediately prior to the intervention (baseline) and at the end of the intervention.

During the first week of the study, mothers followed their normal diet (Figure 1). After assessment of baseline habitual diet, mothers commenced a 2-week dietary intervention. Diets during this period aimed to reduce intakes of discretionary foods, saturated fats and added sugars in comparison with the women’s habitual diet. To increase participant compliance, all meals and snacks were provided to mothers *via* a home delivery service from Lite n’ Easy, Queensland, Australia. All meals were designed to contain healthy amounts of fat and sugar according to Food Standards Australia New Zealand, and to meet the energy and nutritional requirements for lactating women (Food Standards Australia New Zealand, 2019). Home visits were conducted weekly during the dietary intervention phase to collect infant stool samples, inquire about any issues, and to undertake anthropometric measures on mothers and their infants.

This study was part of a larger study (Leghi et al., 2021), which aimed to determine the effect of a reduced fat and sugar maternal dietary intervention on HM production, and the associated macronutrient (lactose, protein, and fat) and metabolic hormone (insulin, leptin, and adiponectin) profiles.

### Maternal and infant anthropometric measures

Maternal and infant anthropometric data were collected at enrolment, immediately prior to the dietary intervention, and at the conclusion of the dietary intervention. Maternal weight was measured using calibrated electronic scales (accurate to 0.1 kg). Maternal height was measured using a Stadiometer (accurate to 0.01 m). Maternal body composition was measured using bioimpedance spectroscopy (Impedimed SFB-7). Infant weight was measured using calibrated electronic scales (Medela Baby Scales, accurate to 2 g). Infant length was measured using a Stadiometer (accurate to 0.01 m). Head circumference was measured using flexible, non-stretchable measuring tape with increments of 0.1 cm and checked against a static measure. Infant body composition was measured using bioimpedance spectroscopy (Impedimed SFB-7).

### Maternal dietary assessment

Baseline dietary intake (for 1 week prior to the dietary intervention) was assessed using the Automated Self-Administered 24-Hour Dietary Recall (ASA24) system (Subar et al., 2012). Mothers completed three online 24-h dietary recalls: two on weekdays and one on a weekend day. During the intervention period, mothers were asked to keep a food diary and to record any foods or drinks that they consumed other than those provided, as well as any food that they did not consume from the provided meals and snacks. Maternal energy and macronutrient intakes during the dietary intervention phase were analysed using FoodWorks 10; Xyris Software (Table 1). Baseline dietary intake data were missing for one mother-infant dyad. The relative difference of individual dietary factors between pre-and post-diet was calculated using the following formula: week 3 intake (g)–week 1 intake (g)/week 3 intake (g) × 100.

**Table 1:** Estimation of maternal dietary factors before and during the dietary intervention.

Maternal ID	Week 1 (pre-diet intervention)					Week 2					Week 3				
	Protein (g)	Fat (g)	Saturated fat (g)	Sugar (g)	Fibre (g)	Protein (g)	Fat (g)	Saturated fat (g)	Sugar (g)	Fibre (g)	Protein (g)	Fat (g)	Saturated fat (g)	Sugar (g)	Fibre (g)
1	75.68	93.25	36.61	51.69	21.48	98.90	49.97	18.77	574.40	142.11	88.24	53.29	16.08	74.08	28.94
2	102.02	124.75	52.93	93.09	20.54	92.76	49.40	16.65	76.40	35.21	97.89	51.11	15.93	77.35	32.79
3	83.87	96.54	34.64	122.42	25.30	89.10	53.46	15.78	84.18	33.60	82.26	48.85	15.14	82.41	33.40
4	93.41	152.42	62.97	130.91	34.70	100.10	52.42	16.33	86.29	35.74	104.13	59.12	19.53	94.57	37.94
5	138.26	128.60	47.10	135.07	34.20	99.40	54.00	16.08	74.69	33.57	89.79	49.10	13.30	79.32	34.73
6^a^						90.06	43.72	17.67	82.14	28.70	81.50	46.39	16.34	79.09	26.02
7	121.53	155.86	70.22	194.14	32.59	94.32	54.95	19.55	92.18	31.43	92.82	48.53	17.38	80.70	30.63
8	97.79	98.39	34.18	97.22	28.16	89.13	55.70	20.28	86.78	42.06	85.99	54.00	16.66	80.86	30.49
9	127.52	189.62	72.56	135.01	47.95	104.35	59.40	16.80	80.23	34.24	94.62	56.43	15.77	88.15	35.22
10	69.73	119.17	52.92	147.00	28.82	92.86	48.28	14.99	86.21	34.74	91.99	47.55	15.76	85.12	32.76

a The three baseline 24-h dietary recalls were not completed by this mother.

### Infant stool sample collection

Participants serve as their own controls. Infant stool samples were collected immediately before and after the intervention. Due to variations in infant bowel habits, some samples were collected before or after the intended sampling day. Pre-diet samples were collected up to 3 days before, while for post-diet samples, all were collected within 2 days prior to 5 days after the intended sampling day, with the exception of one infant stool sample that was collected 11 days after (Table 2).

**Table 2:** Infant stool sample collection times across the study.

Infant ID	Pre-diet collection time	Post-diet collection time
1	Day 7	Day 20
2	Day 7	Day 20
3	Day 5	Day 20
4	NA	Day 20
5	Day 5	Day 20
6	Day 4	Day 23
7	Day 7	Day 32
8	Day 5	Day 22
9	Day 4	Day 26
10	Day 6	Day 26

Participants self-collected infant stool samples by taking E-swabs of diapers. Stool swabs in 1 ml liquid Amies media were stored in the participant’s home freezer (-20°C) before being collected at the next home visit and transferred to the laboratory where they were defrosted and vortexed for 5 s to release the sample from the swab into the liquid transport medium. Samples were then aliquoted and stored at −800°C until analysis.

### DNA extraction and metagenomic analysis

DNA was extracted from pre-and post-diet stool samples using the QIAamp 96 PowerFecal QIAcube HT Kit (Qiagen) on the QIAcube HT system (Qiagen). The resulting DNA was quantitated using a high sensitivity dsDNA fluorometric assay (QuantIT, ThermoFisher, Q33120). Samples needed to reach a minimum of 0.2 ng/μl to pass quality control requirements. Libraries were prepared using a modified protocol, using the Illumina® DNA Prep, (M) Tagmentation (96 Samples) kit (Illumina #20018705), to allow for reduced reaction volumes. Libraries were indexed with IDT® for Illumina Nextera DNA Unique Dual Indexes Set A-D (Illumina #20027213–16). Pooled libraries were prepared for sequencing on the NovaSeq6000 (Illumina) with 2 × 150 bp paired-end chemistry. Sequencing was performed to a target depth of 3 Gbp (2 Gbp minimum, approximately 7–16 M paired-end reads) raw read generation before quality filtering. Data quality was guaranteed at 75% and above for reads >Q30 at the completion of the sequencing run. All sample preparation and sequencing was performed at Microba Life Sciences Limited.^1^

### Metagenomic data processing

#### Metagenomic sequencing data quality control

Shotgun metagenomic sequencing data quality control was performed at Microba Life Sciences Limited (see footnote 1). Paired-end DNA sequencing data were demultiplexed and adaptor trimmed using Illumina BaseSpace Bcl2fastq2 (v2.20) accepting one mismatch in index sequences. Reads were then quality trimmed and residual adaptors removed using the software Trimmomatic v0.39 (Bolger et al., 2014) with the following parameters: -phred33 LEADING:3 TRAILING:3 SLIDINGWINDOW:4:15 CROP:100000 HEADCROP:0 MINLEN:100. Human DNA was identified and removed by aligning reads to the human genome reference assembly 38 (GRCh38.p12, GCF_000001405) using bwa-mem v0.7.17 (Li, 2013) with default parameters except minimum seed length set to 31 (−k 31). Human genome alignments were filtered using SAMtools v1.7 (Li et al., 2009), with flags-ubh -f1-F2304. Any read pairs where at least one read mapped to the human genome with >95% identity over >90% of the read length were flagged as human DNA and removed. All samples were then randomly sub-sampled to a standard depth of 14 M reads, which was then rarefied to 11 M reads.

#### Quantification of microbial species, gene and pathway abundances

Species profiles were obtained with the Microba Community Profiler v2.0.2 (Parks et al., 2021) using the Microba Genome Database (MGDB) v2.0.0 as the reference. Reads were assigned to genomes within MGDB, and the relative cellular abundance of species clusters was estimated and reported. Quantification of gene and pathway abundance in the metagenomic samples was performed using the Microba Gene and Pathway Profiler (MGPP) v0.1.0 against the Microba Genes (MGENES) database v2.0.0. MGPP is a two-step process. In step 1, all open reading frames (ORFs) from all genomes in MGDB were clustered against UniRef90 (Suzek et al., 2015) release 2019/09 using 90% identity over 80% of the read length with MMSeqs2 Release 10-6d92c (Steinegger and Söding, 2018). Gene clusters were then annotated with the UniRef90 identifiers and linked to the Enzyme Commission (accessed *via* UniProt 2019/09) and Transporter Classification Database (Saier et al., 2016) annotations *via* the UniProt ID mapping service.^2^ Enzyme Commission annotations were used to determine the encoding of MetaCyc (Caspi et al., 2020) pathways in each genome using enrichM^3^ and pathways that were complete or near complete (completeness >80%), were classified as encoded. In step 2, all DNA sequencing read pairs that aligned with one or more bases to the gene sequence from any protein within an MGENES protein cluster were summed. DNA sequence reads were aligned directly to genome sequences. The genome sequences were annotated using full length ORFs, and the coordinates of these ORFs/genes recorded. The genes were annotated using the entire protein and clustered into protein clusters. When a DNA sequence read aligned to a genome, we required a minimum of one base overlap of the read to its’ proximal annotated ORF, in order to count the read toward that protein sequence. These counts were then aggregated for each gene cluster. In essence, we relied on the specificity of the DNA. Read alignments were resolved to a single alignment for each read when possible. We typically were able to assign on average 85% of all reads in a sample. Any unresolved (multi mapped reads) were discarded. In this way, reads were counted only once. Abundances of encoded pathways of species reported as detected by MCP were calculated by averaging the read counts of all genes for each enzyme in that pathway. There was no taxonomy associated with the gene clusters.

#### Antibiotic resistance genes

Assembled reads were aligned against sequences from known antibiotic resistance genes (ARGs) using ABRicate^4^ and starAMR.^5^To ensure accuracy, the Resfinder database and comprehensive antibiotic resistance database (CARD) were utilized in the search.

### Statistical analyses

Univariate statistical tests were applied to a filtered set of features. In general, different criteria were applied for taxonomic data and functional data. For pre- *vs* post-diet comparison analyses, paired *t*-tests were used for comparisons of paired measurements of square root-transformed microbial and gene abundance data. Rare taxonomic reads present in less than three samples and low abundance reads with a mean relative abundance within infants positive for that taxa of less than 0.5% were excluded. Rare functional reads present in less than three samples and low abundance reads with a maximum sample count less than 2 were excluded. Pearson correlation tests were used to compare continuous variables for analyses of associations between the difference in individual maternal dietary factors and square root-transformed microbial and gene abundance data. Square root transformation was used to normalize data distribution. Rare taxonomic features (species) where read counts were 0 in all samples and low abundance features with a maximum sample count of less than 100 reads were excluded. Rare functional features where read counts were 0 in all samples and low abundance features with a maximum sample count of less than two reads were excluded. *p* values were corrected for multiple hypothesis testing using the Benjamini and Hochberg false discovery rate correction. Corrected values of *p* < 0.05 are considered statistically significant. No confounders were included in the analysis due to the within individual design and the homogeneity of the group. Redundancy Analysis (RDA) was used to visualize relationships between samples in two-dimensions for identifying sample clusters based on the maternal fiber, protein, sugar, and fat intake.

## Results

### Participants

Maternal and infant characteristics of the study participants are shown in Table 3.

**Table 3:** Participant characteristics (*n* = 10).

Variable	*N*% or Mean (Range) Pre-diet intervention	*N*% or Mean (Range) Post-diet intervention
Maternal age (years)	31.5 [22–37]	31.6 [22–38]
Infant age (months)	3.2 months [1.8–4.9]	4 months [2.5–5.8]
Maternal BMI, kg/m^2^	24.9 [17–32.9]	24.5 [16.9–32.77]
BMI category:		
Normal (18.5–24.9)	3 (30%)	4 (40%)
Overweight (25–29.9)	4 (40%)	3 (30%)
Obesity class I (30–34.9)	1 (10%)	1 (10%)
Underweight (<18.5)	2 (20%)	2 (20%)
Maternal probiotic use^a^	1 (10%)	1 (10%)
Infant solid use	0 (0%)	0 (0%)
Mode of delivery:		
Vaginal	6 (60%)	
Emergency Caesarean section	2 (20%)	
Elective Caesarean section	2 (20%)	
Gestational age (weeks)	39.4 [38–41]	
Male infants	6 (60%)	

a one or two doses (not often).

### Effect of the dietary intervention on maternal dietary factor intake and body composition

**Figure 2:**
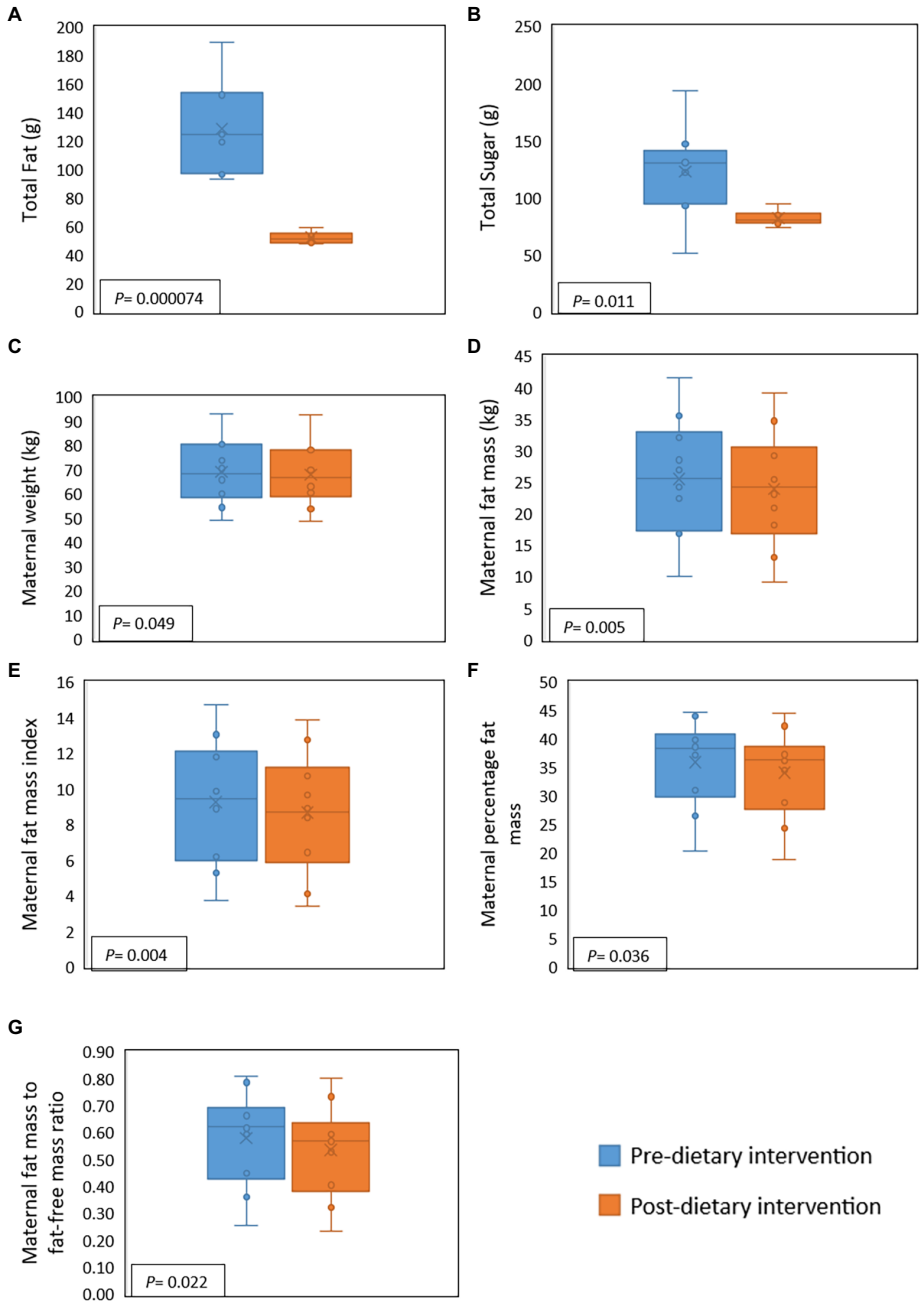
Effect of a 2-week maternal dietary intervention on maternal dietary intakes and body composition. Total maternal fat intake **(A)** and total maternal sugar intake **(B)** were significantly reduced by the dietary intervention. Maternal weight **(C)**, fat mass **(D)**, fat mass index **(E)**, percentage of fat **(F)**, and fat mass to fat-free mass ratio **(G)** were also significantly decreased after the dietary intervention. *X* represents the mean value, while the solid line represents the median.

During the intervention, compared to baseline values, maternal fat, saturated fat, and sugar intake decreased significantly by 59.6, 67.5, and 32.9%, respectively. However, for one participant sugar intake increased during the intervention (Figure 2). There were, however, no significant differences detected for maternal protein and fiber intake. Several changes in maternal body composition were also identified post-intervention (Figure 2), with significant reductions in maternal weight (*p* = 0.049), maternal fat mass (*p* = 0.005), fat mass index (*p* = 0.004), percentage of fat mass (*p* = 0.036), and maternal fat mass to fat-free mass ratio (*p* = 0.022).

### Pre- *vs* post-diet infant gut microbiome composition

**Figure 3:**
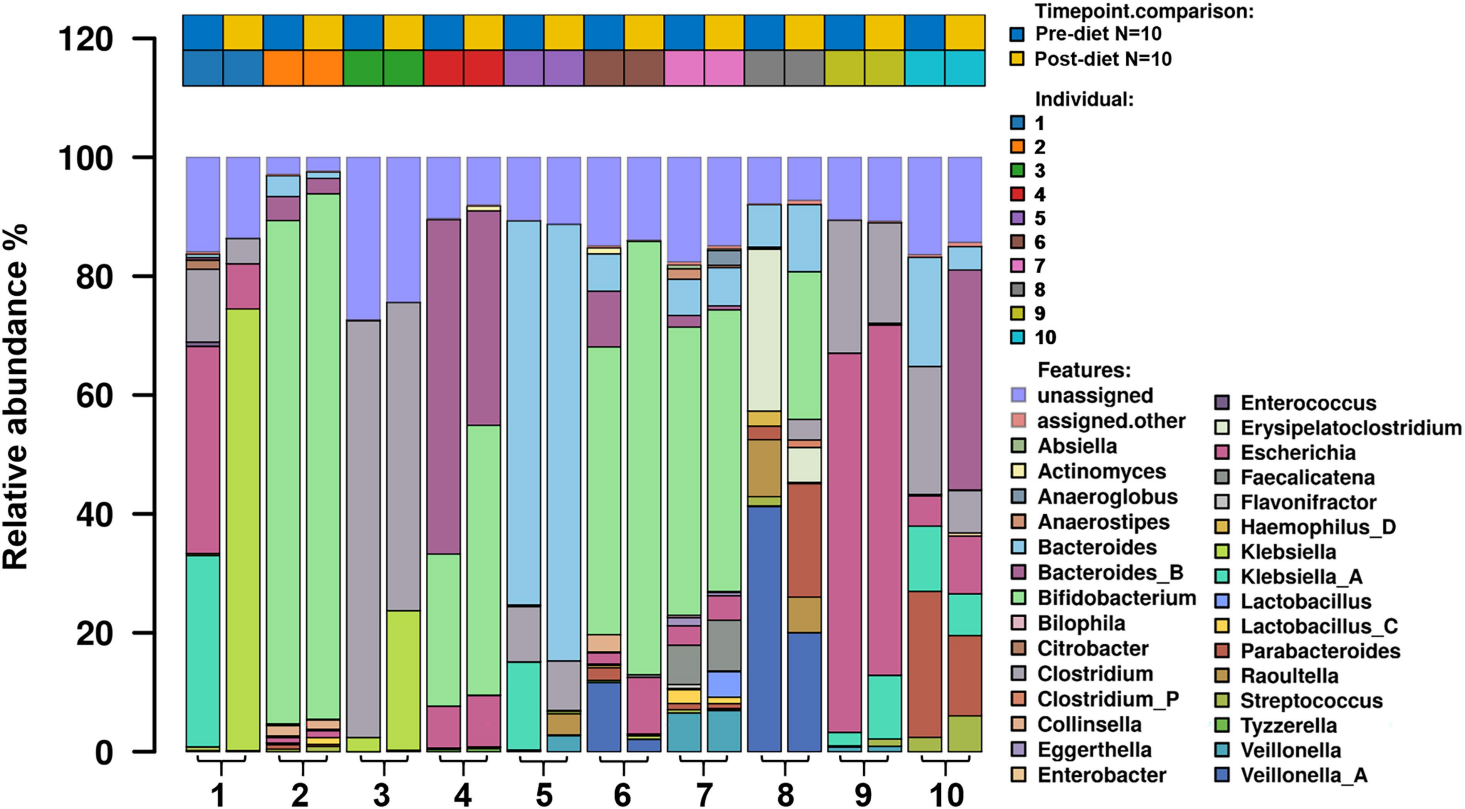
The relative abundance of bacterial genera in the infant gut microbiome pre- and post-dietary intervention. Only the top 30 most abundant genera are shown.

**Figure 4:**
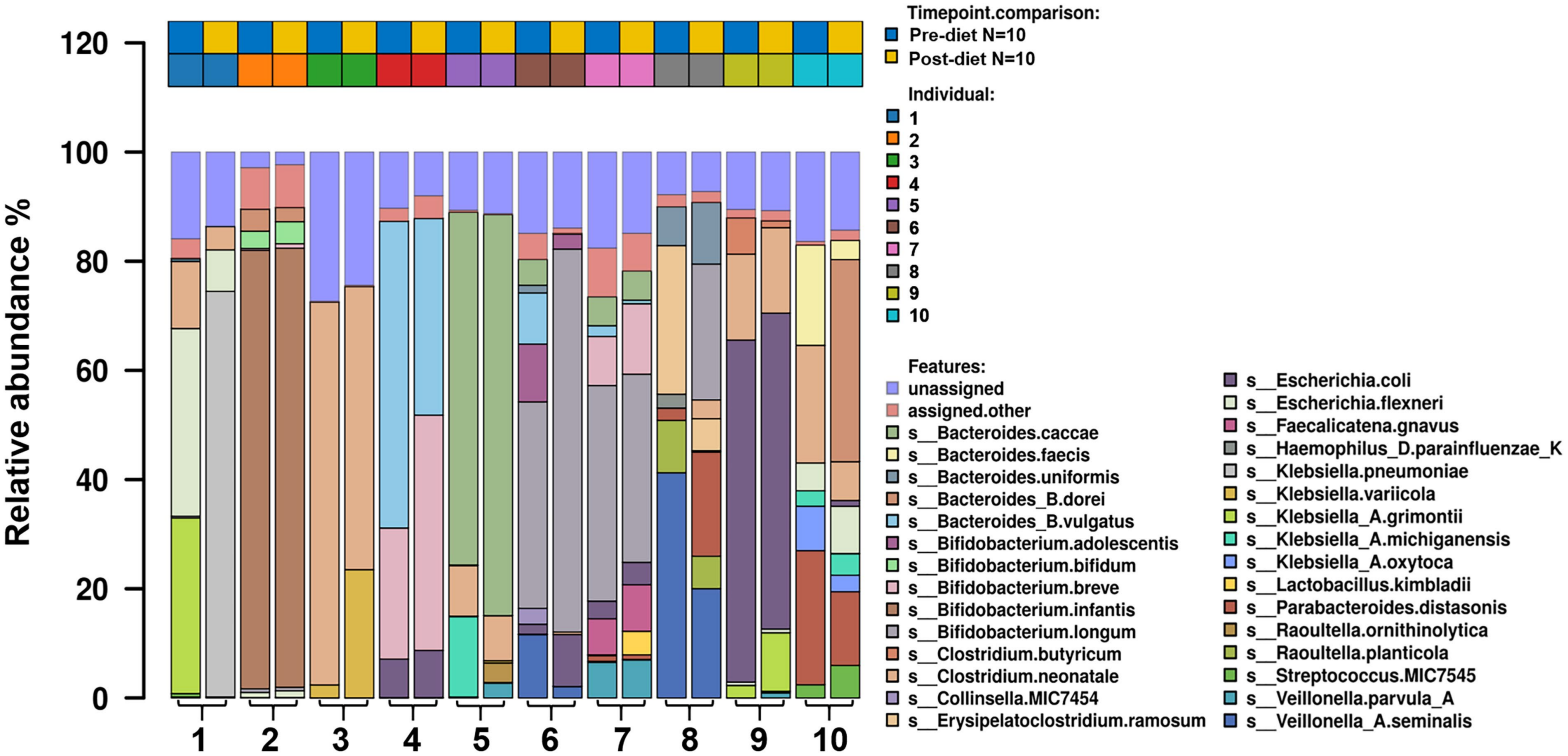
The relative abundance of bacterial species in the infant gut microbiome pre- and post-dietary intervention. Only the top most abundant species are shown.

Number of reads that passed QC as well as the number of reads that mapped for each of the protocols for all samples are reported in (Supplementary Table 1). The most abundant bacterial genera identified in stool samples were *Bifidobacterium* spp. (24.3%), *Bacteroides* spp. (17.5%), and *Clostridium* spp. (11.5%; Figure 3). At the species level, these were *Clostridium neonatale* (11%), *Bifidobacterium longum* (10.4%), and *Bifidobacterium infantis* (8.04%; Figure 4). Seven *Bifidobacterium* spp. were identified, with high inter-individual variability in their abundance. The highest mean abundances were for *B. longum* (10.4%), *B. infantis* (8.04%), and *Bifidobacterium breve* (4.5%). *Bifidobacterium infantis* was present in only one infant stool sample (at 80.3% relative abundance) and its’ relative abundance did not change after the dietary intervention (80.4%). Other identified *Bifidobacterium* spp. were *Bifidobacterium bifidum*, *Bifidobacterium dentium*, *Bifidobacterium adolescentis*, and *Bifidobacterium animalis*, with relative abundances ranging from 0.05 to 10.6% and being present in both pre- and post-diet samples of at least one infant.

**Figure 5:**
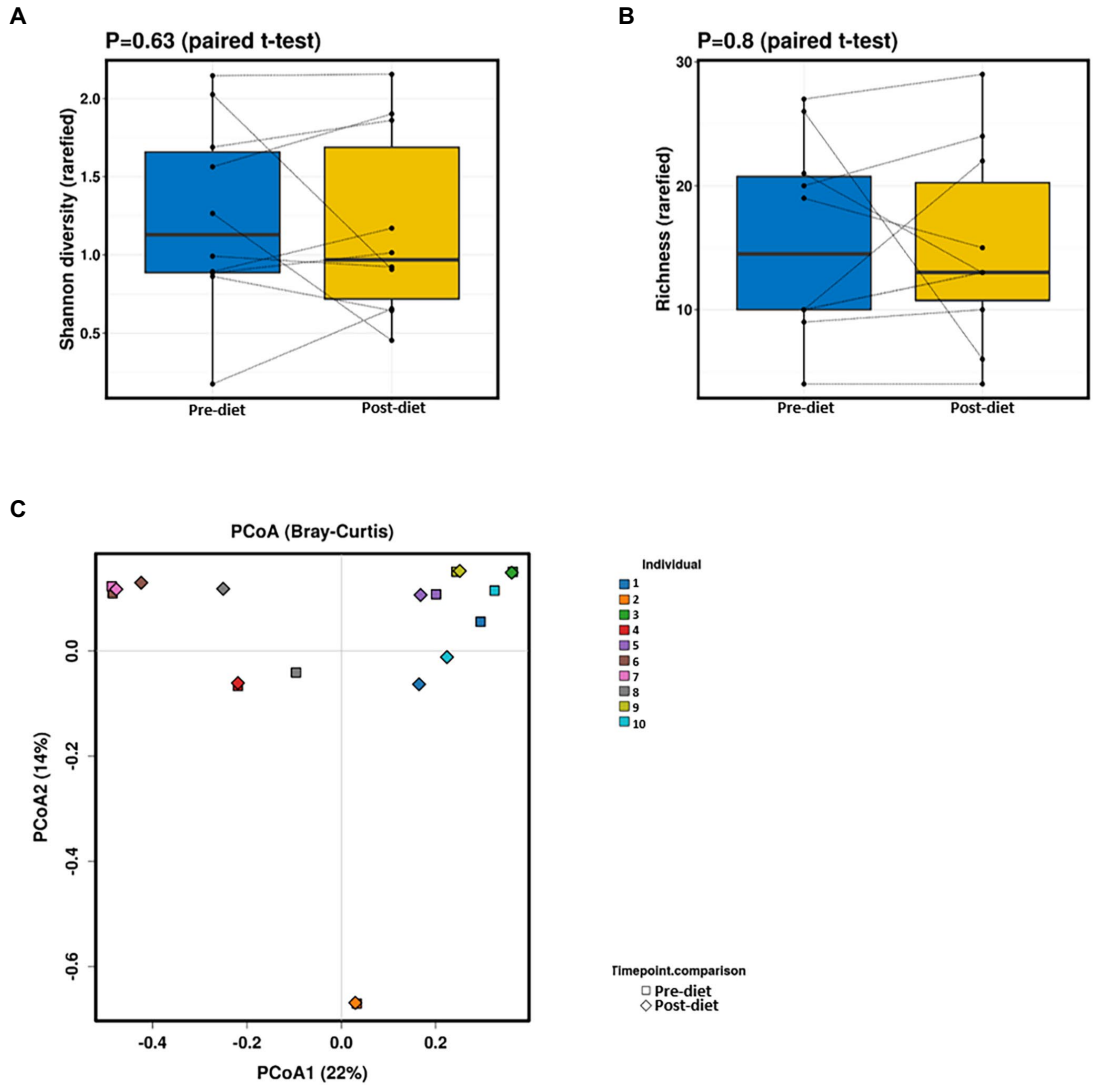
No significant differences in infant gut alpha-diversity [Shannon diversity index **(A)** or richness **(B)**] were detected between pre- and post-diet samples (blue and yellow, respectively). Principal coordinates analysis (PCoA) **(C)** of Bray–Curtis distances showed no significant differences between pre-diet and post-diet samples.

Pre- and post-diet samples were statistically compared using paired *t*-tests to identify any differences in infant gut microbial composition within each infant. No statistically significant differences were identified in the relative abundance of any bacterial species between pre-*vs* post-diet samples, potentially due to the low participant numbers and high level of inter-individual variation. However, some microbial compositional changes were identified within each infant. For example, *B. breve* was present in three pre-diet samples (0.35, 24, and 8.9%), and its relative abundance increased in post-diet samples (0.82, 43.1, and 12.9%). It is also worth noting that one infant, whose mother had an increased sugar intake during the intervention, showed the greatest difference in gut microbiome composition between pre- and post-diet, with decreased relative abundance of *Klebsiella grimontii* (32.2 vs. 0%), *Escherichia flexneri* (34.4 vs. 7.6%), and *Clostridium neonatale* (12.3 vs. 4.3%), and increased relative abundance of *Klebsiella pneumoniae* (0.3 vs. 74.3%; Figure 4). There was no difference in infant gut microbiome alpha-diversity in pre-*vs* post-diet samples (Figures 5A,B; richness *p* = 0.08; Shannon index *p* = 0.63). Nor was there any difference in Bray–Curtis distances between pre-*vs* post-diet samples (Figure 5C; Adonis, *p* = 0.99).

### Pre- *vs* post-diet infant gut microbiome functional potential

**Figure 6:**
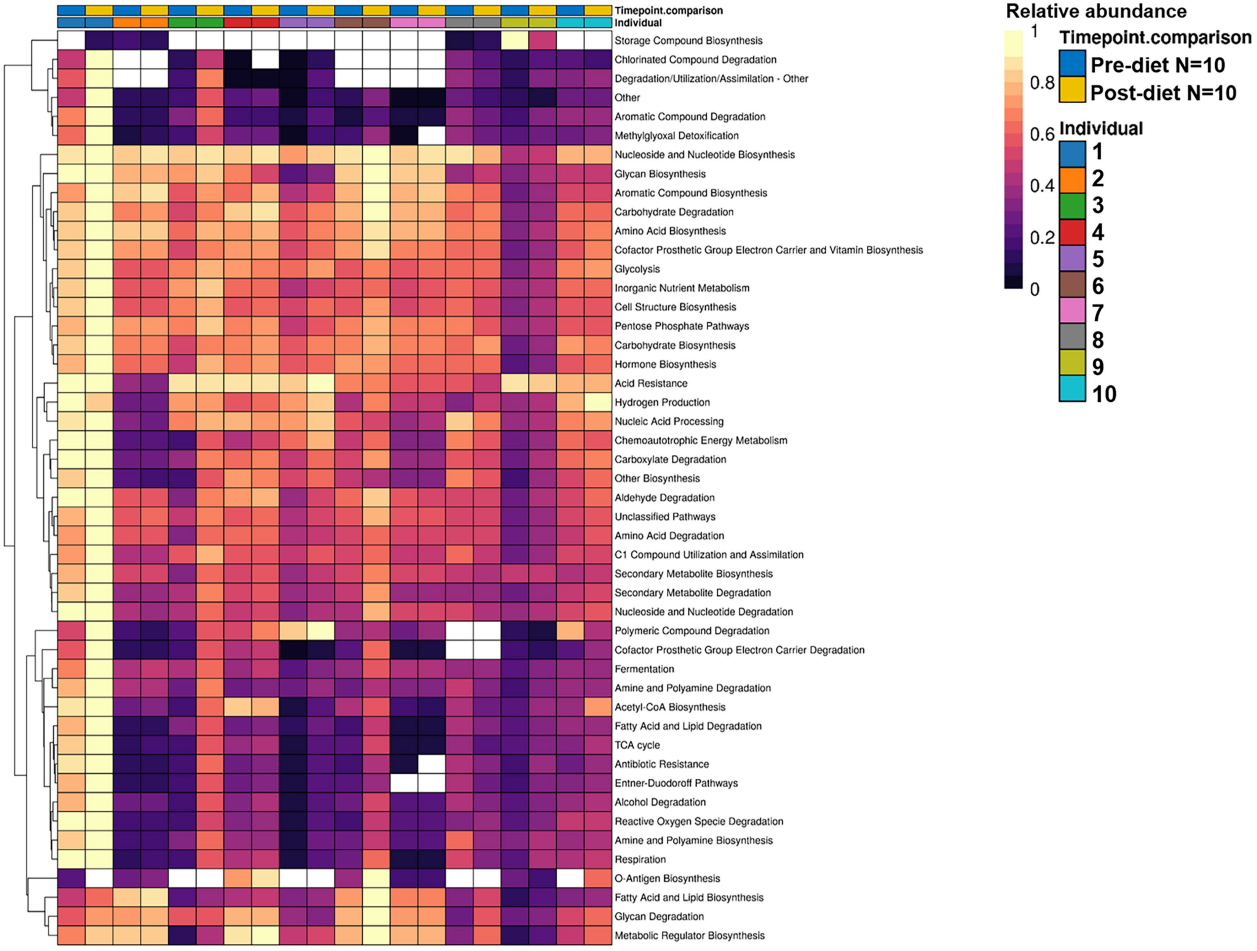
Hierarchically clustered heatmap showing the differential relative abundance of bacterial functional metabolic pathways across pre-diet vs. post-diet infant stool samples. A significant increase in the relative abundances of genes involved in 28 bacterial metabolic pathways was detected in post-diet compared to pre-diet samples.

While post-diet samples did not differ statistically from pre-diet samples in terms of microbial composition, alterations were identified in the functional potential of these microbial communities, with significant differences in multiple bacterial metabolic pathways detected. Overall, 808 gene clusters were significantly different between pre- and post-diet samples (Supplementary Table 2). Significant increases in the abundances of genes involved in 28 bacterial metabolic pathways were detected (Table 4). For instance, post-diet samples showed a significant increase in the potential for biosynthesis of co-factor prosthetic group electron carriers and vitamins (*p* = 0.003), metabolic regulators (*p* = 0.003), amino acids (*p* = 0.005), aromatic compounds (*p* = 0.008), carbohydrates (*p* = 0.01), and fatty acids and lipids (*p* = 0.01). However, while all gene clusters and metabolic pathways that were significantly different post-intervention were rendered insignificant after adjustment for FDR. *p*-values ranged from 0.06 to 0.078, indicating a strong trend toward significance (Table 4). The infant whose mother had an increased sugar intake during the dietary intervention showed the most substantial difference in gut microbiome functional potential between pre-and post-diet samples, with a general increase in the relative abundance of genes involved in most functional metabolic pathways (Figure 6).

**Table 4:** Significantly different bacterial metabolic pathways identified in infant stool samples pre- and post-diet intervention (calculated using paired *t*- test).

Function	p-value	FDR
Cofactor prosthetic group electron carrier and vitamin biosynthesis	0.003	0.066
Metabolic regulator biosynthesis	0.003	0.066
Amino acid biosynthesis	0.005	0.066
Unclassified pathways	0.008	0.066
Aromatic compound biosynthesis	0.008	0.066
Carbohydrate biosynthesis	0.009	0.066
Fatty acid and lipid biosynthesis	0.01	0.066
Carbohydrate degradation	0.01	0.066
Aldehyde degradation	0.01	0.072
Glycolysis	0.01	0.072
Amino acid degradation	0.01	0.072
Fermentation	0.01	0.072
Reactive oxygen species degradation	0.02	0.072
Secondary metabolite degradation	0.02	0.072
Inorganic nutrient metabolism	0.02	0.072
Secondary metabolite biosynthesis	0.02	0.072
Hormone biosynthesis	0.02	0.072
Alcohol degradation	0.02	0.073
Glycan degradation	0.03	0.078
Cofactor prosthetic group electron carrier degradation	0.03	0.078
Entner–Doudoroff pathways	0.03	0.078
TCA cycle	0.04	0.078
Cell structure biosynthesis	0.04	0.078
Antibiotic resistance	0.04	0.078
Pentose phosphate pathways	0.04	0.078
Nucleoside and nucleotide biosynthesis	0.04	0.078
Carboxylate degradation	0.04	0.078
Fatty acid and lipid degradation	0.04	0.078

The detected changes in functional potential may possibly be explained by changes in the taxonomical composition of infant stool samples between pre- and post-diet. The dietary intervention resulted in an increase in the mean abundances of *Bacteroides dorei* (0.4 vs. 4%), B. breve (3.3 vs. 5.7%), *B. longum* (7.8% vs. 13%), and *Klebsiella variicola* (0.24 vs. 2.4%), and a decrease in the mean abundances of *Bacteroides vulgatus* (6.8 vs. 3.7%), *B. adolescentis* (1.1 vs. 0.28%), *C. neonatale* (13 vs. 9.1%), *E. flexneri* (4.1 vs. 1.8%), and *K. grimontii* (3.5 vs. 1.1%). The functional potential of these organisms are shown in (Table 5).

**Table 5:** Significantly different bacterial metabolic pathways identified in infant stool samples pre- and post-dietary intervention and the corresponding organisms that potentially account for the functional changes.

Function	Corresponding organisms
Co-factor prosthetic group electron carrier and vitamin biosynthesis	*Bacteroides dorei, Bacteroides vulgatus, Bifidobacterium adolescentis, Bifidobacterium breve, Bifidobacterium longum, Clostridium neonatale, Escherichia flexneri, Klebsiella variicola,* and *Klebsiella grimontii*
Metabolic regulator biosynthesis	*B. dorei, B. vulgatus, B. adolescentis, B. breve, B. longum, C. neonatale, E. flexneri, K. variicola,* and *K. grimontii*
Amino acid biosynthesis	*B. dorei, B. vulgatus, B. adolescentis, B. breve, B. longum, C. neonatale, E. flexneri, K. variicola,* and *K. grimontii*
Aromatic compound biosynthesis	*B. dorei, B. vulgatus, B. adolescentis, B. breve, B. longum, C. neonatale, E. flexneri, K. variicola,* and *K. grimontii*
Carbohydrate biosynthesis	*B. dorei, B. vulgatus, B. adolescentis, B. breve, B. longum, C. neonatale, E. flexneri, K. variicola,* and *K. grimontii*
Fatty acid and lipid biosynthesis	*B. dorei, B. vulgatus, B. adolescentis, B. breve, B. longum, C. neonatale, E. flexneri, K. variicola,* and *K. grimontii*
Carbohydrate degradation	*B. dorei, B. vulgatus, B. adolescentis, B. breve, B. longum, C. neonatale, E. flexneri, K. variicola,* and *K. grimontii*
Aldehyde degradation	*B. dorei, B. vulgatus, B. adolescentis, B. breve, B. longum, C. neonatale, E. flexneri, K. variicola,* and *K. grimontii*
Glycolysis	*B. dorei, B. vulgatus, B. adolescentis, B. longum, C. neonatale, E. flexneri, K. variicola,* and *K. grimontii*
Amino acid degradation	*B. dorei, B. vulgatus, B. adolescentis, B. breve, B. longum, C. neonatale, E. flexneri, K. variicola,* and *K. grimontii*
Fermentation	*B. dorei, B. adolescentis, B. vulgatus, B. longum, C. neonatale, E. flexneri, K. variicola,* and *K. grimontii*
Reactive oxygen species degradation	*B. dorei, B. vulgatus, C. neonatale, E. flexneri, K. variicola,* and *K. grimontii*
Secondary metabolite degradation	*B. dorei, B. vulgatus, C. neonatale, E. flexneri, K. variicola,* and *K. grimontii*
Inorganic nutrient metabolism	*B. dorei, B. vulgatus, B. adolescentis, B. breve, B. longum, C. neonatale, E. flexneri, K. variicola,* and *K. grimontii*
Secondary metabolite biosynthesis	*B. dorei, B. vulgatus, B. adolescentis, B. breve, B. longum, C. neonatale, E. flexneri, K. variicola,* and *K. grimontii*
Hormone biosynthesis	*None*
Alcohol degradation	*B. dorei, B. vulgatus, B. adolescentis, B. breve, B. longum, C. neonatale, E. flexneri, K. variicola,* and *K. grimontii*
Glycan degradation	*B. dorei, B. adolescentis, B. breve, B. longum, C. neonatale, E. flexneri, K. variicola,* and *K. grimontii*
Co-factor prosthetic group electron carrier degradation	None
Entner–Doudoroff pathways	*E. flexneri, K. variicola,* and *K. grimontii*
TCA cycle	*C. neonatale, E. flexneri,* and *K. grimontii*
Cell structure biosynthesis	*B. dorei, B. vulgatus, B. adolescentis, B. breve, B. longum, C. neonatale, E. flexneri, K. variicola,* and *K. grimontii*
Antibiotic resistance	*E. flexneri, K. variicola,* and *K. grimontii*
Pentose phosphate pathways	*B. adolescentis, B. vulgatus, B. longum, E. flexneri, K. variicola,* and *K. grimontii*
Nucleoside and nucleotide biosynthesis	*B. dorei, B. vulgatus, B. adolescentis, B. breve, B. longum, C. neonatale, E. flexneri, K. variicola,* and *K. grimontii*
Carboxylate degradation	*B. dorei, B. vulgatus, B. adolescentis, B. breve, B. longum, C. neonatale, E. flexneri, K. variicola,* and *K. grimontii*
Fatty acid and lipid degradation	*B. dorei, B. vulgatus, B. breve, C. neonatale, E. flexneri, K. variicola,* and *K. grimontii*

### Maternal dietary factors and infant gut microbiome composition and function

Although infant gut microbiome composition did not differ significantly in pre-*vs* post-diet samples, correlations were identified between changes in individual dietary factors throughout the dietary intervention, the abundance of certain bacterial taxa, and the functional potential of the associated infant gut microbiome. We calculated the difference in individual dietary factors between pre-and post-diet as relative difference (delta). One mother-infant dyad was excluded from this analysis due to a lack of baseline dietary intake data.

#### Fiber

**Figure 7:**
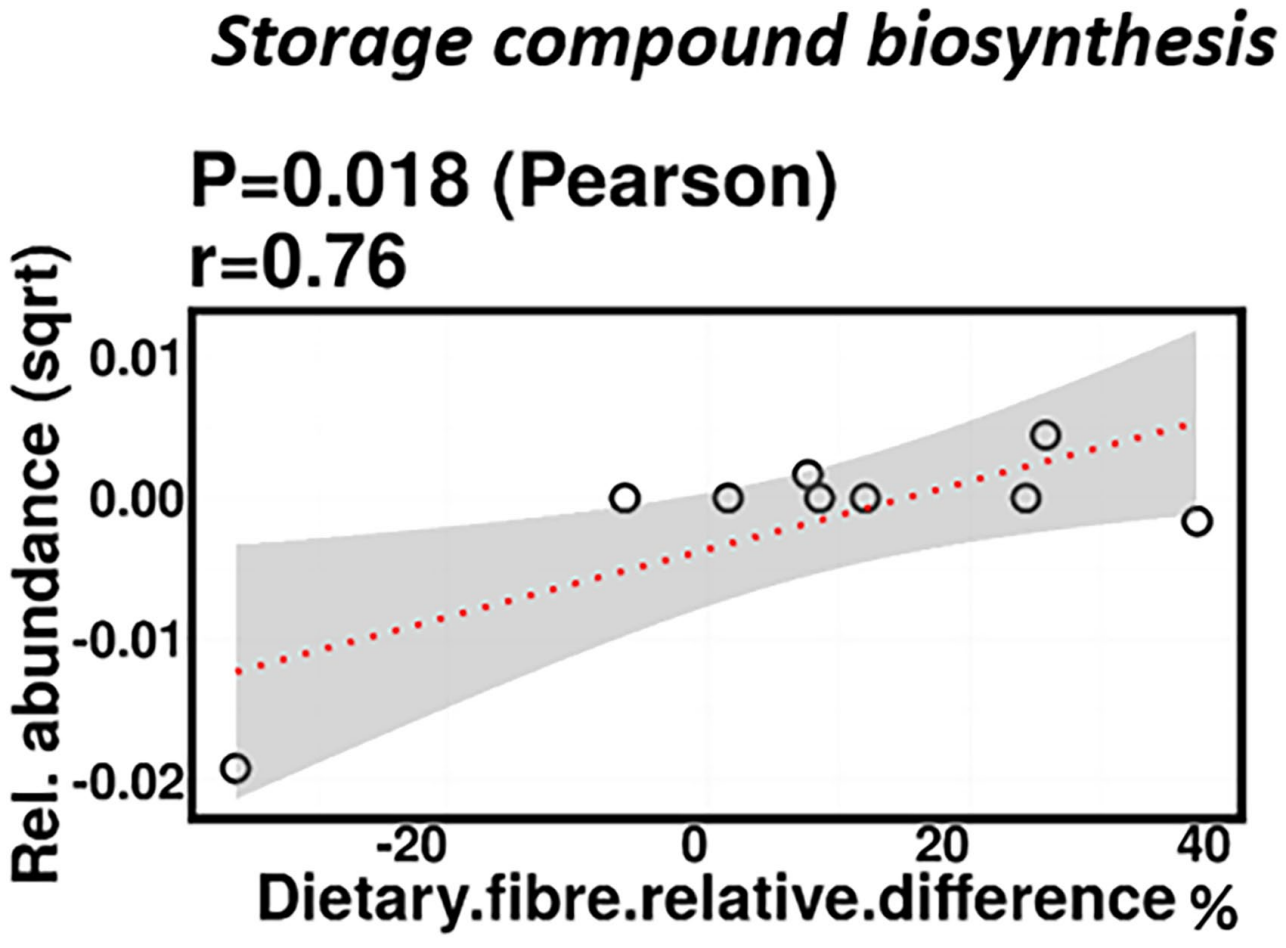
Change in maternal fiber intake % was positively correlated with the abundance of genes involved in storage compound biosynthesis.

No significant correlations were identified between relative difference in dietary fiber intake and infant gut microbiome composition. At a functional level, relative difference in dietary fiber intake was correlated with significant changes in 28 gene clusters in the infant gut microbiome (Supplementary Table 3). An increased abundance of genes involved in the storage compound biosynthesis metabolic pathway were observed (*p* = 0.018; Figure 7). Interestingly, the abundance of the gene for the enzyme responsible for cyanophycin synthesis (cyanophycin synthase) was positively correlated with the relative difference in dietary fiber (*p* = 0.025). Cyanophycin acts as a temporary nitrogen reserve and accumulates in the form of granules in the cytoplasm during phosphate or sulfur starvation (Ziegler et al., 2002).

#### Protein

**Figure 8:**
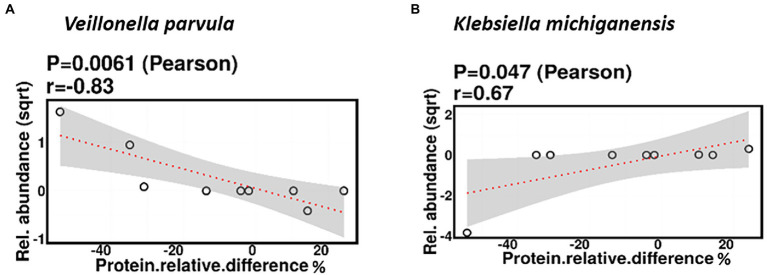
Change in maternal protein intake % was negatively correlated with the relative abundance of *Veillonella parvula* **(A)** and positively correlated with the relative abundance of *Klebsiella michiganensis* **(B)**.

Relative difference in dietary protein content was negatively correlated with the relative abundance of *Veillonella parvula* (mean relative abundance 2.16%, *p* = 0.006), while positively correlated with the relative abundance of *Klebsiella michiganensis* (mean relative abundance 3.58%, *p* = 0.047; Figure 8). Functionally, relative difference in dietary protein was correlated with significant changes in 51 gene clusters (Supplementary Table 3). However, no significant correlations were identified with any bacterial metabolic pathways.

#### Sugar

**Figure 9:**
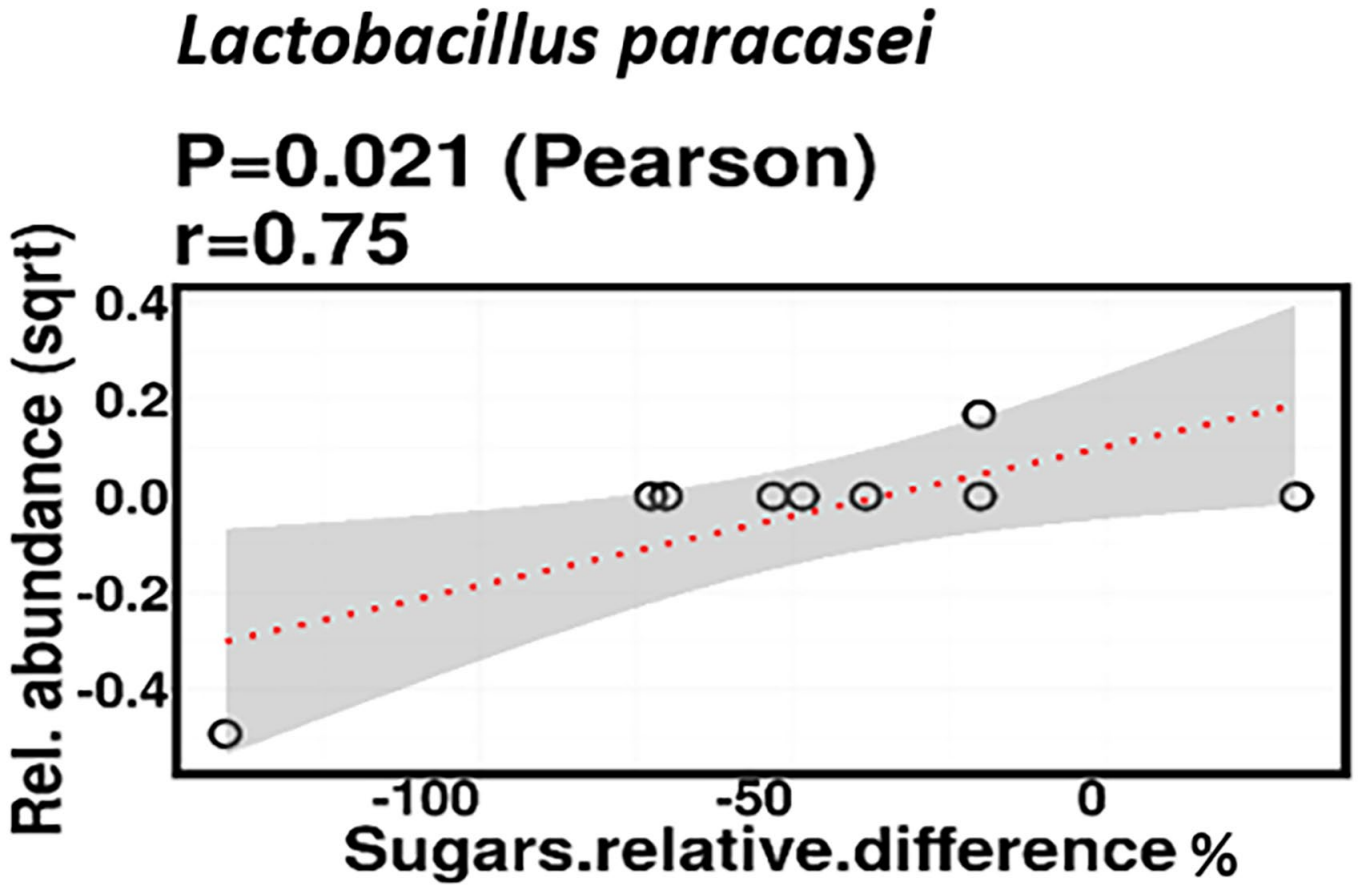
Change in maternal sugar intake % was positively correlated with the relative abundance of *Lactobacillus paracasei*.

**Figure 10:**
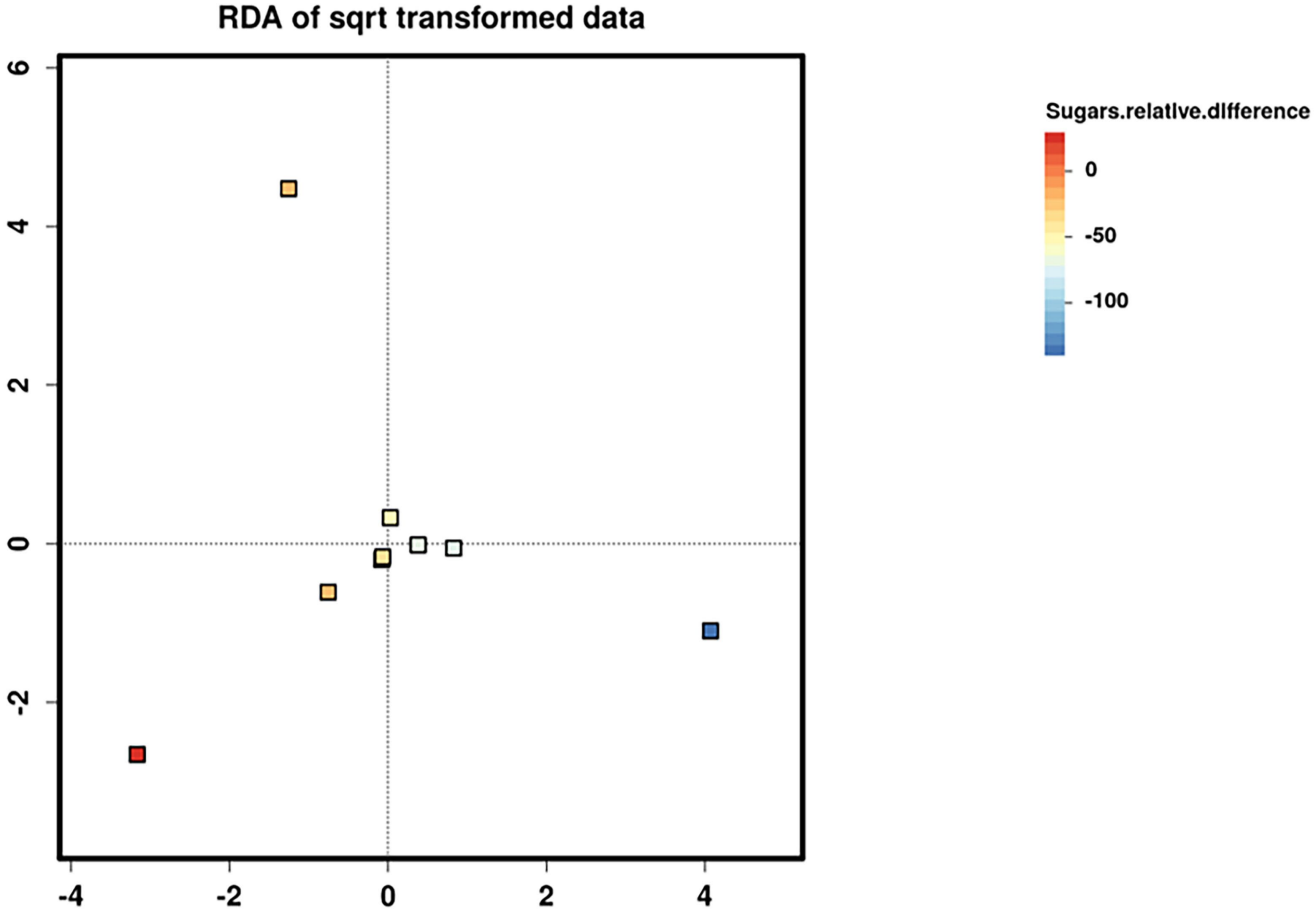
Redundancy analysis (RDA) biplots showing the two first axes of ordination for nine infant stool microbiome samples. Samples are coloured according to the change in maternal sugar intake with the dietary intervention.

**Figure 11:**
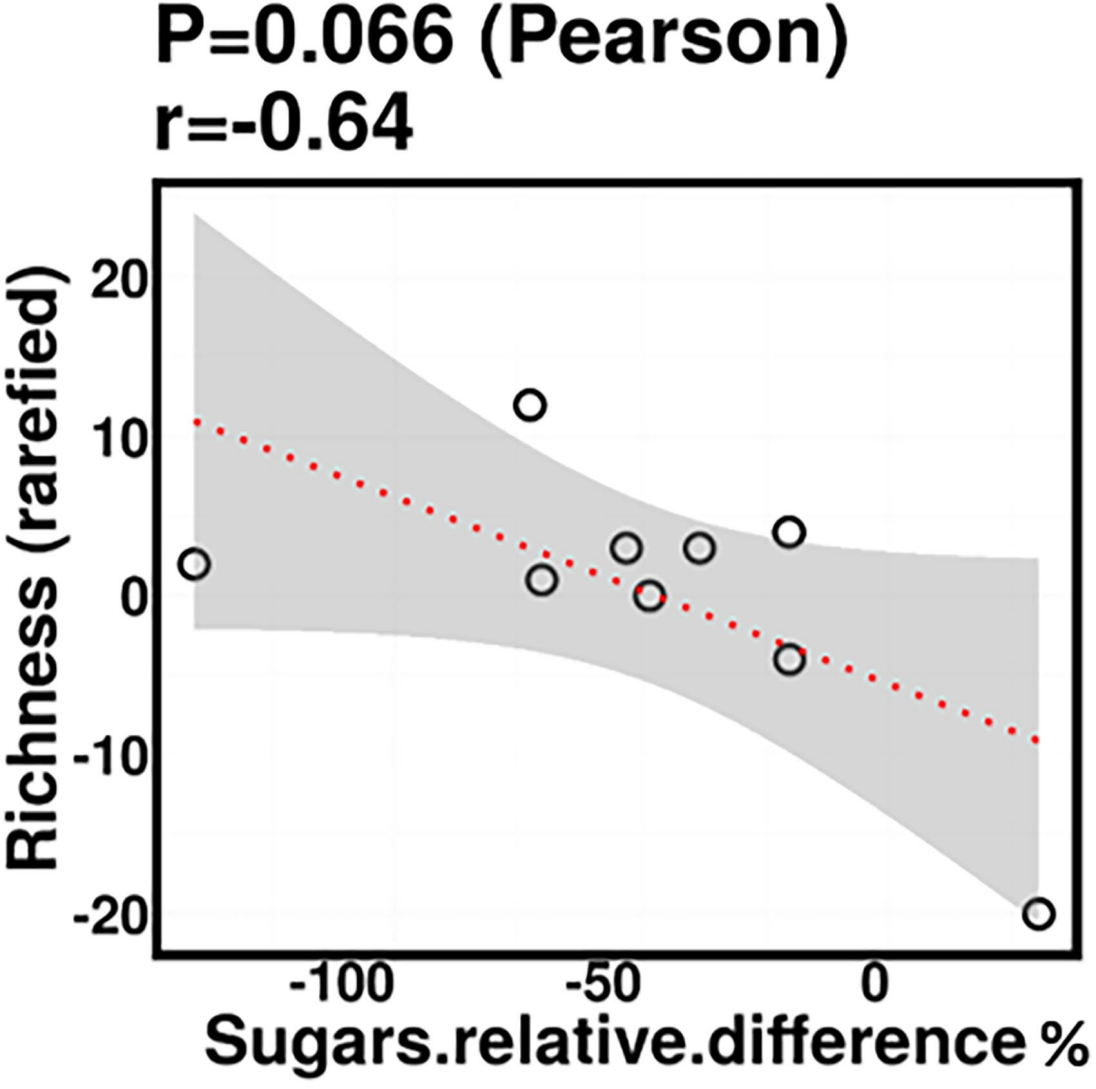
Infant stool bacterial richness was negatively correlated with change in maternal sugar intake with the dietary intervention; however, this was not statistically significant.

Relative difference in dietary sugar was positively correlated with the relative abundance of *Lactobacillus paracasei* (mean relative abundance 0.83%, *p* = 0.021; Figure 9). Interestingly, the RDA showed significant clustering of infant gut bacterial communities according to change in relative difference in dietary sugar (*p* = 0.046; Figure 10). In addition, a downward trend (although not significant) was observed in the bacterial richness of infant stool samples and increased relative difference in dietary sugar (*p* = 0.06; Figure 11). Relative difference in dietary sugar was correlated with significant changes in 150 gene clusters in the infant gut microbiome (Supplementary Table 3). However, no significant correlations were identified for any bacterial metabolic pathways.

#### Fat

**Figure 12:**
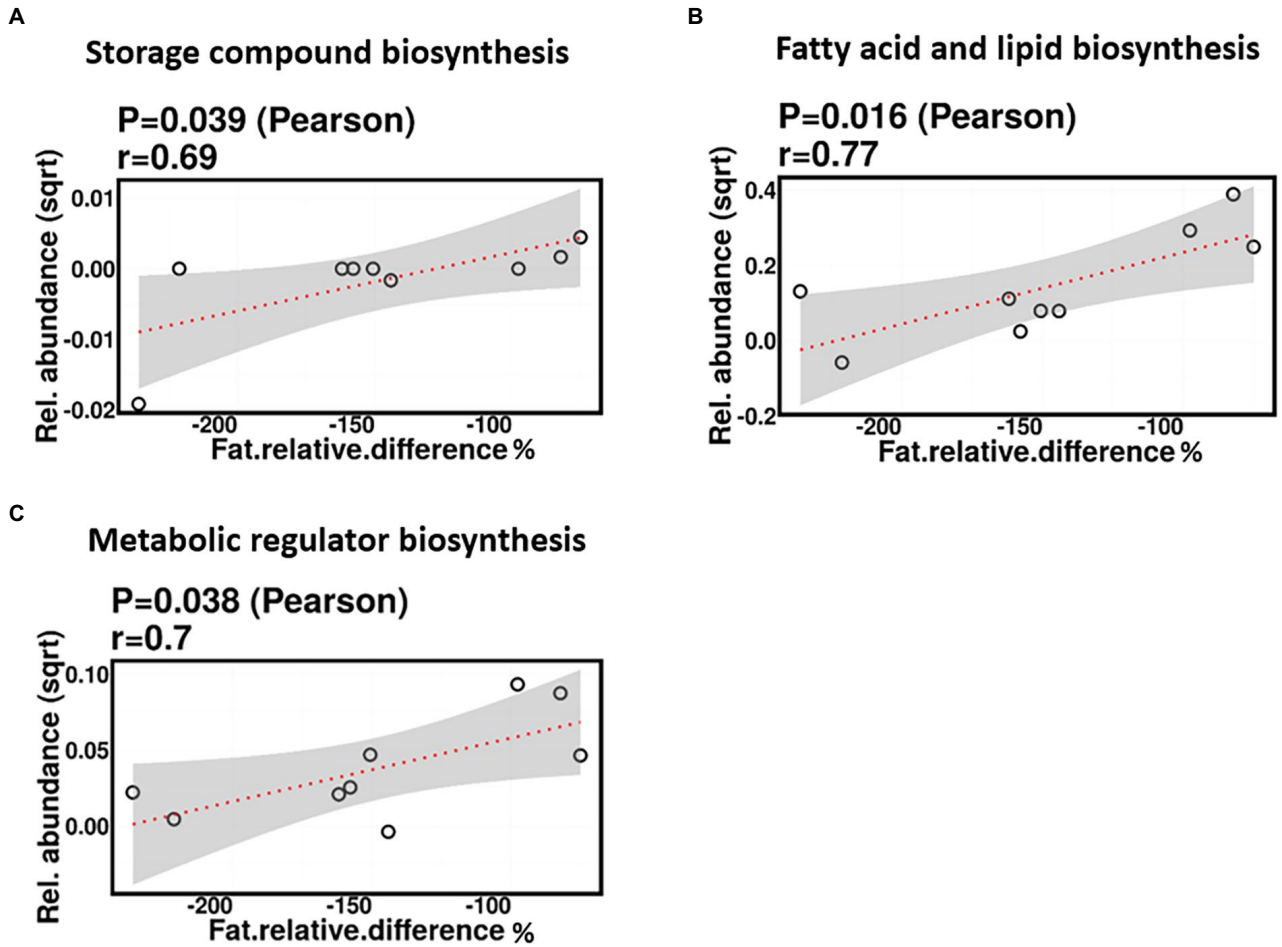
Change in maternal fat intake was positively correlated with the abundance of genes involved in storage compound biosynthesis **(A)**, fatty acid and lipid biosynthesis **(B)**, and metabolic regulator biosynthesis **(C)**.

No significant correlations were identified between relative difference in dietary fat intake and infant gut microbiome composition. However, associations were identified between relative difference in dietary fat and the functional potential of the infant gut microbiome. Relative difference in dietary fat was correlated with significant changes in 140 gene clusters (Supplementary Table 3). These gene clusters fell into three metabolic pathways: storage compounds biosynthesis, fatty acid and lipid biosynthesis, and metabolic regulator biosynthesis, all of which were positively correlated with the relative difference in dietary fat (*p* = 0.039, 0.016, and 0.038, respectively; Figure 12).

### Antibiotic resistance genes

**Figure 13:**
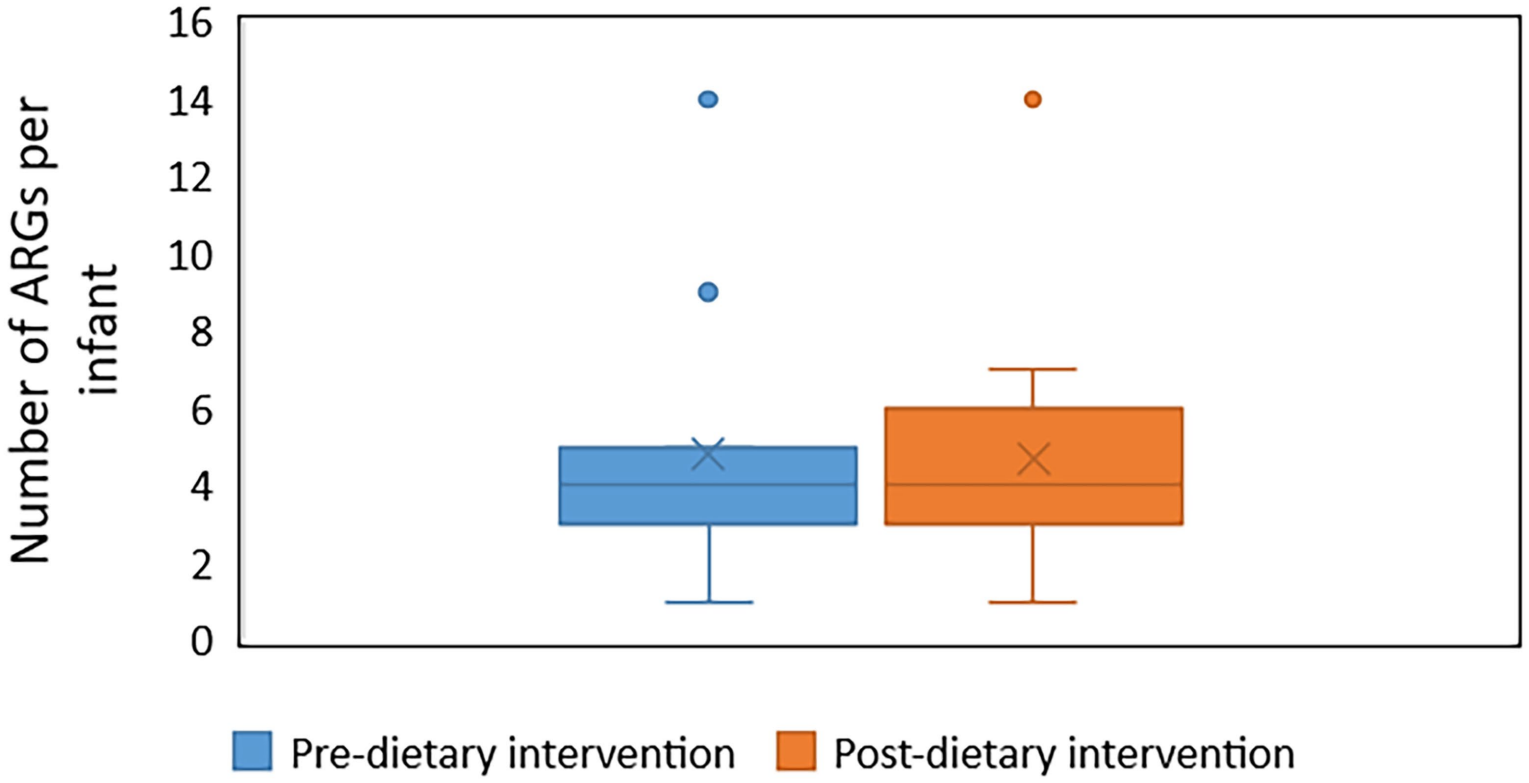
The number of antibiotic resistance genes (ARGs) in infant stool samples collected pre-and post-dietary intervention. X represents the mean value.

Forty unique ARGs were detected in the infant gut microbiome across the pre-and post-diet samples. For both pre-and post-diet samples, ARGs were found across eight different antibiotic classes. The mean number of ARGs per infant in pre-and post-diet samples was 4.8 and 4.7, respectively (Figure 13). The most common ARGs identified in pre-diet samples potentially conferred resistance to tetracycline, while the most common in post-diet samples potentially conferred resistance to tetracycline, erythromycin, and azithromycin (Table 6). Overall, the most common ARGs in both pre-and post-diet samples were tet(Q), tet(O), and msr(D) (Table 6).

**Table 6:** Most commonly detected antibiotic resistance genes and their associated antibiotic class in pre-and post-dietary intervention infant faecal samples.

Antibiotic resistance genes	Antibiotic class	Related pathogens	Frequency within samples	
			Pre-diet intervention	Post-diet intervention
*mef*(A)	Erythromycin, azithromycin	*Streptococcus pneumoniae*	2	2
*blaTEM*-1B	Ampicillin	*Escherichia coli*	2	2
*blaOXY*-6-2	Ampicillin	*Klebsiella oxytoca*	2	1
*tet*(Q)	Tetracycline	*Butyrivibrio fibrisolvens*	6	4
*tet*(W)	Tetracycline	*Bacteroides fragilis*	2	2
*aph*(3′)-Ia	Kanamycin	*E. coli*	2	0
*cfx*A4	Ampicillin	*B. fragilis*	2	2
*blaACI*-1	Ampicillin	*Acidaminococcus intestini*	2	2
*cfxA3*	Ampicillin	*Pseudomonas aeruginosa*	2	0
*tet*(O)	Tetracycline	*Campylobacter coli* or *Campylobacter jejuni*	3	2
*erm*(F)	Erythromycin, azithromycin	*B. fragilis*	1	2
*msr*(D)	Erythromycin, azithromycin	*S. pneumoniae*	1	3

## Discussion

We show that a maternal dietary intervention consisting of pre-prepared reduced fat and sugar meals during lactation for 2 weeks significantly alters the functional potential of the infant gut microbiome. The dietary intervention did not, however, affect the bacterial composition of the infant gut microbiome. In addition, changes in individual dietary factors over the course of the diet were correlated with the abundance of certain bacterial taxa, as well as the functional potential of the infant gut microbiome.

The dietary intervention resulted in detection of an increased abundance of genes involved in 28 bacterial metabolic pathways. These metabolic pathways are involved in the biosynthesis and degradation of co-factors, prosthetic groups, electron carriers, vitamins, amino acids, fatty acids, lipids, carbohydrates, and secondary metabolites. Previous studies have shown that infant diet modulates the functional capacity of the infant gut microbiome (Stewart et al., 2018) and that maternal diet modulates the functional capacity of the HM microbiome (Seferovic et al., 2020). For example, Stewart et al. reported that breast milk intake (partially or exclusively) was significantly associated with increased lipid and carbohydrate metabolic pathways in the infant gut microbiome and that breast milk was the single strongest factor responsible for modulation of the infant gut microbiome (Stewart et al., 2018). In comparison, Seferovic et al. showed that a maternal dietary intervention in lactating women can modify the functional capacity of the HM microbiome (Seferovic et al., 2020). A high-fat vs. a high-carbohydrate diet and a high-glucose vs. a high-galactose diet were associated with significant increases in multiple bacterial metabolic pathways (many of them involved in amino acid biosynthesis) in HM. However, similar to our study, the taxonomic composition of the HM microbiome was minimally affected by the maternal dietary intervention. The small sample size in our study may have reduced the power to detect an effect of maternal diet on HM microbiome composition; therefore, further studies with a larger cohort and a longer duration of dietary intervention may reveal more effects of maternal diet that were not detectable in our study. The significant change in the infant gut microbiome function is unlikely driven by the macronutrients level in HM, as the dietary intervention did not change fat, protein, and lactose concentrations in HM (Leghi et al., 2021). Thus, this change might be driven by other HM biochemical components such as human milk oligosaccharides (HMOs), which have been shown to alter the functional capacity of the HM microbiome (Seferovic et al., 2020).

To our knowledge, no previous study has investigated the effects of maternal dietary intervention during lactation on the functional potential of the infant gut microbiome in humans. Studies in pregnant and lactating mice have reported that consumption of a high-fat diet is associated with changes in offspring gut microbiome function (Wankhade et al., 2017; Srinivasan et al., 2018); however, no studies have looked at the effects of a low-fat diet on offspring gut microbiome function. Srinivasan et al. reported that a maternal high-fat diet before, during, and after pregnancy is associated with increased pathways involved in fructose and mannose metabolism and decreased pathways related to indole alkaloid biosynthesis, α-linolenic acid metabolism, and carotenoid metabolism in the offspring gut microbiome (Srinivasan et al., 2018), while Wankhade et al. reported that high-fat diet consumption by dams during pregnancy and lactation was associated with an increase in pathways involved in regulating microbial replication and repair in the offspring gut microbiome (Wankhade et al., 2017). In contrast, our study showed that a reduced fat and sugar maternal dietary intervention resulted in increased presence of pathways involved in genetic material synthesis, such as nucleoside and nucleotide biosynthesis and cell structure biosynthesis. It should be noted, however, the functional inference reported in the above animal studies (Wankhade et al., 2017; Srinivasan et al., 2018), was generated using PICRUSt (phylogenetic investigation of communities by reconstruction of unobserved states), a computational tool for indirect analysis of function predicted from 16S rRNA gene sequencing. This method is somewhat limited in that it is not actually detecting the presence of microbial genetic material in samples, beyond the 16S rRNA gene, but instead inferring function based on taxonomy. This type of functional inference is thereby not as accurate as metagenomic data.

The significant increase in the abundance of genes involved in biosynthesis and degradation of vitamins, amino acids, fatty acids, lipids, and carbohydrates that we observed after the intervention is in agreement with the known role of the gut microbiome in the human metabolism of these dietary components (Rowland et al., 2018; Schoeler and Caesar, 2019). Several gut bacterial species have been associated with amino acid and carbohydrate metabolism and short chain fatty acid (SCFA) synthesis. In addition, the human gut microbiome plays an important role in synthesizing vitamins, especially those that humans cannot synthesize, such as thiamin (vitamin B1; LeBlanc et al., 2013). Indeed, after the intervention, there was a significant increase in the abundance of genes involved in the thiamine diphosphate biosynthesis I pathway (*p* = 0.028), responsible for thiamin synthesis. The mechanism by which the maternal dietary intervention generated these alterations is unclear, since no significant changes in the infant gut microbiome composition were detected after the intervention, and HM macronutrient content was not affected by the intervention (Leghi et al., 2021). However, there were clear compositional differences in the microbiome between pre-and post-diet samples for one individual, whose mother interestingly had an increase in sugar intake during the intervention. Increased sugar intake may have changed a certain component in HM such as HMOs (Seferovic et al., 2020), which could drive this marked change in the infant gut microbiome composition. Future research should consider increasing the sample size and investigating potential associations with HMO profile to validate these findings.

We also identified correlations between the relative change in individual dietary factors and the abundance of genes involved in some bacterial metabolic pathways. Relative difference in dietary fibre was correlated with a significant increase in genes associated with the storage compound biosynthesis metabolic pathway. No such finding has been reported by previous studies investigating the maternal dietary effect on the HM and infant gut microbiomes. However, only one study has linked total maternal fibre intake with changes in the beta diversity of the overall composition of HM KEGG bacterial metabolic pathways (LeMay-Nedjelski et al., 2020). Additionally, relative difference in dietary fat was correlated with a significant increase in genes associated with three bacterial metabolic pathways: storage compounds, fatty acid and lipid, and metabolic regulatory compounds biosynthesis. These results are consistent with those from a previous study in mice, where a maternal high-fat diet was reported to increase bacterial metabolic pathways involved in lipid metabolism and bile acid secretion synthesis of the offspring gut microbiome function in a sex-specific manner (Wankhade et al., 2018). Other studies of non-lactating mice reported that high-fat diet consumption is associated with a significant increase in the abundance of genes associated with fatty acid metabolism (Xiao et al., 2017) and fatty acid biosynthesis in the gut microbiome compared to a high-sugar diet (Shan et al., 2019) and control diet (Shang et al., 2017). In contrast to our findings, Hildebrandt et al. reported that when mice on a standard chow diet switched to a high-fat diet, they showed an increased abundance of genes involved in signal transduction and membrane transport and a decreased abundance of genes associated with amino acid and carbohydrate metabolism (Hildebrandt et al., 2009). The biological significance of these findings is unknown; however, it has been reported that bacteria can store the excess of certain nutrients, including lipids, in the form of storage granules as a source of metabolic precursors or as an energy reserve (Murphy and Vance, 1999). Collectively, our results show that maternal diet during lactation influences the infant gut microbiome functional capacity; however, further studies are needed to investigate the mechanisms driving these changes and whether these changes have positive or negative health effects.

The 2-week maternal dietary intervention had no statistically significant effect on the composition of the infant gut microbiome. This is in agreement with the results of the only previous study to investigate the association between maternal diet during lactation and the infant gut microbiome composition (Babakobi et al., 2020). This finding is also in agreement with other dietary interventional studies, where short-term dietary interventions either did not induce changes or failed to significantly alter the gut microbiome composition in healthy adults (Korem et al., 2017), adults at high risk for developing metabolic disorders (Roager et al., 2019), children (Shulman et al., 2017), and mice (Dimova et al., 2017). However, given the high level of inter-individual variation in the infant gut microbiome, the low participant numbers in our study may have limited our ability to detect statistically significant changes in bacterial composition.

We also identified significant correlations between relative differences in individual dietary factors and the composition and functional potential of the infant gut microbiome. These appear to be driven by 2–4 participants that exhibited the greatest changes in dietary components. Decreased relative difference in dietary protein was correlated with an increase in the relative abundance of *V. parvula*. This finding is unexpected as *Veillonella* spp. are known for their role in amino acid hydrolysis and fermentation (Dai et al., 2011). However, no previous study has reported such an association. Relative difference in dietary protein was positively correlated with *Klebsiella michiganensis* relative abundance. This finding is in agreement with the amino acid fermenting function of *Klebsiella* spp. (Dai et al., 2011; Cai et al., 2020). However, no previous study has reported such an association. In terms of sugar, decreases in maternal pre- and post-diet sugar levels were associated with decreases in the relative abundance of *L. paracasei*. This finding is consistent with the known sugar metabolizing functions of *Lactobacillus* spp. (Makarova et al., 2006). The positive correlation between *Lactobacillus* spp. abundance and the change in maternal sugar intake is inconsistent with previous studies that associated a high-sugar diet with decreased abundance of *Lactobacillus* spp. in mice (Sen et al., 2017; Yue et al., 2019; Khan et al., 2020). Nevertheless, the increased abundance of *Lactobacillus* spp. in these infants may be beneficial due to the probiotic potential of members of this genus (Martín et al., 2005; Shokryazdan et al., 2014).

The trend toward a negative association between infant stool bacterial richness and relative difference in maternal sugar intake is consistent with observations in mice, wherein mice who consumed a high-sugar diet had decreased alpha-diversity compared to those who consumed a normal diet (Sen et al., 2017; Do et al., 2018). In humans, this finding is in line with those of De Filippo et al., where they showed that gut microbiota richness in African children is higher than European children (De Filippo et al., 2010, 2017). They suggested that the low richness of the gut microbiota in European children could be due to Western diet consumption (high in sugar, animal protein, and fat). However, this finding is based on long-term dietary habits, and there was no direct assessment of dietary intake association with gut microbiome richness. Overall, our results suggest that change in maternal intake of fiber, protein, and sugar during lactation may affect infant gut bacterial composition; however, further studies on a larger cohort need to be conducted to confirm these findings.

Analysis of the infant gut resistome showed that the most prevalent ARGs in pre-and post-diet samples potentially conferred resistance to tetracycline. This finding agrees with previous studies where a high prevalence of resistance to tetracycline was detected in the faecal samples of healthy infants (Gueimonde et al., 2006; Rose et al., 2017; Pärnänen et al., 2018) and those at a high risk of eczema (Loo et al., 2020), suggesting their presence may be unremarkable. The mean number of ARGs per infant did not differ between pre-and post-diet samples (4.8 and 4.7), respectively. In contrast, one study compared the gut resistome of 35 obese children before and after a microbiota-targeted dietary intervention. A diet composed of traditional Chinese medicinal foods, whole grains, and prebiotics resulted in a significant reduction in ARGs within these children (Wu et al., 2016). To date, no study has investigated whether or not maternal diet could affect ARGs of the infant gut microbiome. Infant gut ARGs have been shown to be associated with different factors such as mode of delivery and infant sex (Lebeaux et al., 2021). Maternal diet may also be a factor that could potentially influence the infant gut resistome. Importantly, Pärnänen et al. characterized the HM, infant, and maternal gut microbiomes using metagenomic sequencing in order to identify potential sources of infant gut ARGs. Their results showed that infant gut ARGs resembled those of their own mother’s gut and HM, suggesting vertical transmission from mothers to infants (Pärnänen et al., 2018). If maternal diet can impact the maternal gut resistome, it may, in turn, influence the HM and infant gut resistome. Therefore, future research should consider exploring the relationship between maternal diet and infant gut ARGs in a larger cohort.

## Strengths and limitations

The key strength of this study is the use of a home delivery service to deliver meals to participants, which increases dietary intervention compliance and consistent dietary intake. In addition, the use of shotgun metagenomics allows characterization of functional capacity for the microbial communities. Another strength is that our study design included baseline (pre-diet) samples to establish a baseline microbiome, combined with use of strict inclusion criteria where all participants with factors that have been shown to impact maternal gut, HM, and infant gut microbiomes were excluded, including probiotic and antibiotic use. A number of limitations do, however, need to be acknowledged. The sample size is small, and the duration of the intervention is relatively short; thus, is it unknown whether the same results may be obtained if a similar dietary intervention were applied on a larger cohort and/or for a longer period. Additionally, time of sampling might be a confounding variable, as maternal weight/BMI typically decreases in the early months postpartum, and the infant gut microbiome develops temporally. The effects of other potential confounders such as infant sex and mode of delivery were minimized by including an equal number of these variables. In analyses of relative abundances, every increase or decrease in a certain microbe’s abundance can lead to changes in that from other community members. This is an inherent limitation of using relative abundances to compare between different samples, however, despite this, this method is still well-accepted as an effective way to document the microbial composition of different sample types. Metabolic pathway analyses were not conducted, instead, all reference to these is based on the relative abundance of associated genes, which may or may not have been actively expressed.

## Conclusion

This pilot study is the first to report the effect of a controlled maternal dietary intervention during lactation on the breastfed infant gut microbiome. While the effect of maternal diet during pregnancy on the composition of the infant gut microbiome has been previously investigated (Chu et al., 2016; Lundgren et al., 2018; Savage et al., 2018; Ponzo et al., 2019; Babakobi et al., 2020; García-Mantrana et al., 2020), here, we show that maternal diet during lactation significantly alters the functional potential of the infant gut microbiome. While there were no significant differences in the overall bacterial composition of infant stool samples taken before or after the dietary intervention, we did find associations between changes in individual dietary factor intakes of protein and sugar during lactation and changes in the relative abundances of certain taxa in the infant gut microbiome. The impact of these changes on infant health and development remains to be further investigated. We speculate that the maternal diet may have altered the HM microbiome or other milk components that likely mediated the observed changes in the infant gut microbiome. Future dietary interventional studies investigating the relationship between maternal diet during lactation and the infant gut microbiome should consider examining the HM microbiome and other milk components such as HMOs, antimicrobial peptides, and SCFAs. This may shed light on the mechanism/s by which maternal diet impacts the infant gut microbiome. Results obtained from such studies may allow optimization of dietary recommendations for lactating women to better support breastfed infant health and development.

## Data availability statement

The data presented in the study are deposited in the SRA repository, accession number PRJNA819247.

## Ethics statement

The studies involving human participants were reviewed and approved by The University of Western Australia Human Research Ethics Committee (RA/4/20/4953). The patients/participants provided their written informed consent to participate in this study.

## Author contributions

LS, DG, MW, BM, and MP: study design. AS: sample collection and processing, statistical analyses, and writing original draft. AS, LS, DG, and MP: interpretation. SL: detection of antibiotic resistance genes. GL, MN, LS, DG, MW, BM, and MP: writing review and editing. All authors contributed to the article and approved the submitted version.

## Funding

AS, LS, and DG are supported by an unrestricted research grant from Medela AG, administered by The University of Western Australia. MP is supported by a National Health and Medical Research Council Project grant (APP1144040). Umm Al-Qura University, Saudi Arabia provides a PhD scholarship for AS. The funding bodies were not involved in the design of the study, collection/analysis/interpretation of data, writing of the manuscript, or in the decision to publish the results.

## Conflict of interest

The authors declare that the research was conducted in the absence of any commercial or financial relationships that could be construed as a potential conflict of interest.

## Publisher’s note

All claims expressed in this article are solely those of the authors and do not necessarily represent those of their affiliated organizations, or those of the publisher, the editors and the reviewers. Any product that may be evaluated in this article, or claim that may be made by its manufacturer, is not guaranteed or endorsed by the publisher.

## Footnotes

^1^ www.microba.com/research

^2^ www.uniprot.org/uploadlists/

^3^ https://github.com/geronimp/enrichM

^4^ https://github.com/tseemann/abricate

^5^ https://github.com/phac-nml/staramr
